# Reach adaptation and proprioceptive recalibration following terminal visual feedback of the hand

**DOI:** 10.3389/fnhum.2014.00705

**Published:** 2014-09-08

**Authors:** Victoria Barkley, Danielle Salomonczyk, Erin K. Cressman, Denise Y. P. Henriques

**Affiliations:** ^1^Sensorimotor Control Lab, Centre for Vision Research, Department of Psychology, York UniversityToronto, ON, Canada; ^2^Sensorimotor Control Lab, School of Human Kinetics, University of OttawaOttawa, ON, Canada; ^3^School of Kinesiology and Health Science, York UniversityToronto, ON, Canada

**Keywords:** visuomotor rotation, terminal feedback, motor adaptation, proprioceptive recalibration, vision

## Abstract

We have shown that when subjects reach with continuous, misaligned visual feedback of their hand, their reaches are adapted and proprioceptive sense of hand position is recalibrated to partially match the visual feedback (Salomonczyk et al., 2011). It is unclear if similar changes arise after reaching with visual feedback that is provided only at the end of the reach (i.e., terminal feedback), when there are shorter temporal intervals for subjects to experience concurrent visual and proprioceptive feedback. Subjects reached to targets with an aligned hand-cursor that provided visual feedback at the end of each reach movement across a 99-trial training block, and with a rotated cursor over three successive blocks of 99 trials each. After each block, no cursor reaches, to measure aftereffects, and felt hand positions were measured. Felt hand position was determined by having subjects indicate the position of their unseen hand relative to a reference marker. We found that subjects adapted their reaches following training with rotated terminal visual feedback, yet slightly less (i.e., reach aftereffects were smaller), than subjects from a previous study who experienced continuous visual feedback. Nonetheless, current subjects recalibrated their sense of felt hand position in the direction of the altered visual feedback, but this proprioceptive change increased incrementally over the three rotated training blocks. Final proprioceptive recalibration levels were comparable to our previous studies in which subjects performed the same task with continuous visual feedback. Thus, compared to reach training with continuous, but altered visual feedback, subjects who received terminal altered visual feedback of the hand produced significant but smaller reach aftereffects and similar changes in hand proprioception when given extra training. Taken together, results suggest that terminal feedback of the hand is sufficient to drive motor adaptation, and also proprioceptive recalibration.

## INTRODUCTION

Numerous studies have shown that people can rapidly adapt their reaches when provided with altered visual feedback of their hand, such as a misaligned hand cursor. Moreover, people continue to produce deviated reaches even after the cursor misalignment, or even cursor itself, is removed; such changes in reach direction that are in the opposite direction of the misalignment are known as reach aftereffects (Izawa and Shadmehr, 2011; Taylor and Ivry, 2012; Taylor et al., 2014). Our lab has recently shown that training to reach with distorted visual feedback of the hand also leads to changes in proprioceptive estimates of hand position (Cressman and Henriques, 2009, 2010; Cressman et al., 2010; Salomonczyk et al., 2011; Clayton et al., 2013; Mostafa et al., 2014), such that one perceives their felt hand location to be shifted in the direction consistent with the visuomotor distortion. Moreover, we have shown that it is the discrepancy between vision and proprioception (rather than motor error signals) that drives this change in felt hand position, or what we refer to as proprioceptive recalibration (Henriques and Cressman, 2012). Our aim in this study was to test whether reducing the duration of this discrepancy to only the very end of the reaching movement is sufficient to also lead to changes in felt hand position. That is, we asked whether adapting reaches to terminal feedback of the hand (i.e., to a hand-cursor that appears only at the end of the reach), and hence limiting subjects exposure to a visual-proprioceptive discrepancy, would lead to proprioceptive recalibration.

Reach adaptation following reach training with terminal versus continuous feedback has shown mixed results, with several studies showing poorer reach adaptation following terminal feedback versus continuous feedback (Hinder et al., 2008; Shabbott and Sainburg, 2010; van der Kooij et al., 2013). On the other hand, other studies have found that differences in learning rate or aftereffects produced following training with terminal feedback versus continuous feedback are rather small (Izawa and Shadmehr, 2011; Taylor et al., 2014). In some cases, learning rates have been shown to be comparable, but reach aftereffects are substantially diminished when training feedback is terminal compared to continuous (Hinder et al., 2008; Shabbott and Sainburg, 2010). Inconsistencies across the studies mentioned above may have to do with the size or difficulty of the distortion introduced. In particular, it seems that for more difficult visuomotor rotations (e.g., abruptly introduced or large distortions), aftereffects following training with terminal feedback are either smaller than those with continuous feedback (Shabbott and Sainburg, 2010; van der Kooij et al., 2013; Taylor et al., 2014) or non-existent (Hinder et al., 2008), while introducing the distortion gradually can remove this difference such that aftereffects are even larger (Bernier et al., 2005) or nearly equivalent (Izawa and Shadmehr, 2011) compared to those following continuous feedback training.

Previous results from our lab and others have shown that adaptation to a visuomotor distortion leads to changes not only in hand movement, but also to one’s sense of hand position, or hand motion estimates, when the hand-cursor is visible for most of the reach (Synofzik et al., 2008; Cressman and Henriques, 2009, 2010; Cressman et al., 2010; Izawa and Shadmehr, 2011; Salomonczyk et al., 2011; Izawa et al., 2012; Clayton et al., 2013; Mostafa et al., 2014). Specifically, we have found that after training with a visuomotor distortion, subjects adapt their no cursor reaches (i.e., post-training reaches without visual feedback used to assess reach adaptation), and shift their estimates of the felt position of the reaching hand in the direction consistent with the visual perturbation (Cressman and Henriques, 2009; Cressman et al., 2010; Salomonczyk et al., 2011; Henriques and Cressman, 2012). In accordance with these findings, other studies have also shown that subjects recalibrate their reaches to visual and proprioceptive targets following reach training with laterally displacing prisms (Hay and Pick, 1966; Redding and Wallace, 1996; van Beers et al., 1999; Redding and Wallace, 2000) or with altered visual feedback of the hand in a virtual reality environment (van Beers et al., 2002; Simani et al., 2007). However, it is unclear if these changes in reaches to proprioceptive targets were due to motor adaptation and/or proprioceptive recalibration, as proprioceptive changes were evaluated with goal-directed reaches. Given that changes in goal-directed reaches can be driven by motor adaptation, motor changes may have influenced proprioceptive target localization. To avoid this potential confound between motor adaptation and proprioceptive recalibration, we use a task designed to assess proprioceptive changes independent of motor changes. Specifically, we measure estimates of felt hand position by having a two-joint robot manipulandum precisely place or guide the subject’s hand to a specified location in the workspace, and then ask subjects to judge whether their unseen hand is located to the left or right of either a visual reference marker or the body midline. The extent of change in felt hand position is typically about 20% of the visuomotor distortion, and occurs regardless of whether the distortion is introduced gradually, as in most of our studies (Cressman and Henriques, 2009, 2010; Cressman et al., 2010; Salomonczyk et al., 2011), or abruptly (Salomonczyk et al., 2012). Moreover, this proportional change in felt hand position is evident even when the cursor rotation gradually increases to a maximum of 70° (Salomonczyk et al., 2011). Surprisingly, these changes in perceived hand position are not restricted to changes following reach training with a visuomotor distortion, but have also been found following adaptation to a force-field perturbation (Ostry et al., 2010; Mattar et al., 2013).

Taken together, these results suggest that somatosensory plasticity is an integral part of motor learning, at least when subjects reach with continuous visual feedback of their hand. We have suggested that it is the discrepancy between vision and proprioception that drives perceptual changes of felt hand position (and likely a small portion of the reach aftereffects (Cressman and Henriques, 2010; Henriques and Cressman, 2012). In the current study our goal was to test whether training with gradually introduced terminal feedback, and hence limiting subject’s exposure to a visual-proprioceptive conflict, was sufficient to lead to proprioceptive recalibration. We also wanted to determine how much terminal feedback training was required for both proprioceptive recalibration and reach aftereffects to saturate and potentially achieve levels similar to those seen after continuous feedback training. To do this, we measured open-loop reaching errors and proprioceptive estimates following each of three sets of 99 reach training trials. In the reach training trials, we used a relatively small cursor rotation (30°) that was gradually introduced over 40 trials, as using this type of perturbation should lead to significant reach aftereffects following training with terminal feedback, although possibly smaller than those following continuous feedback. In addition, we had subjects perform several sets of reach training trials in order to investigate whether additional reach training may compensate for possibly slower changes in reach adaptation and/or proprioceptive recalibration. We hypothesize that terminal feedback – although perhaps sufficient to drive reach adaptation – may not induce sizeable proprioceptive recalibration, since subjects see their rotated hand-cursor only at the reach’s endpoint and thus do not have as much exposure to the visual-proprioceptive conflict. Specifically, we predict that any change in felt hand position should be substantially smaller than those produced following training with continuous visual feedback, or at least require additional training to obtain a comparable level of recalibration.

## MATERIALS AND METHODS

### SUBJECTS

Eleven healthy, right-handed adults (mean age = 20.73, SD = 4.45, 7 females) were recruited from York University and volunteered to participate in the current experiment. Prior to participation, subjects were prescreened for self-reported handedness and history of visual, neurological and/or motor dysfunction or injury. In addition to these subjects, the results of ten subjects (mean age = 21.5, SD = 2.62, 5 females) from a previous study (Salomonczyk et al., 2011) were included to serve as a control for comparing the quality of visual feedback on reach adaptation and proprioceptive recalibration. All subjects provided informed consent prior to participating in accordance with the ethical guidelines of York University Human Participants Review Sub-committee.

### GENERAL EXPERIMENTAL SET-UP

**FIGURE 1:**
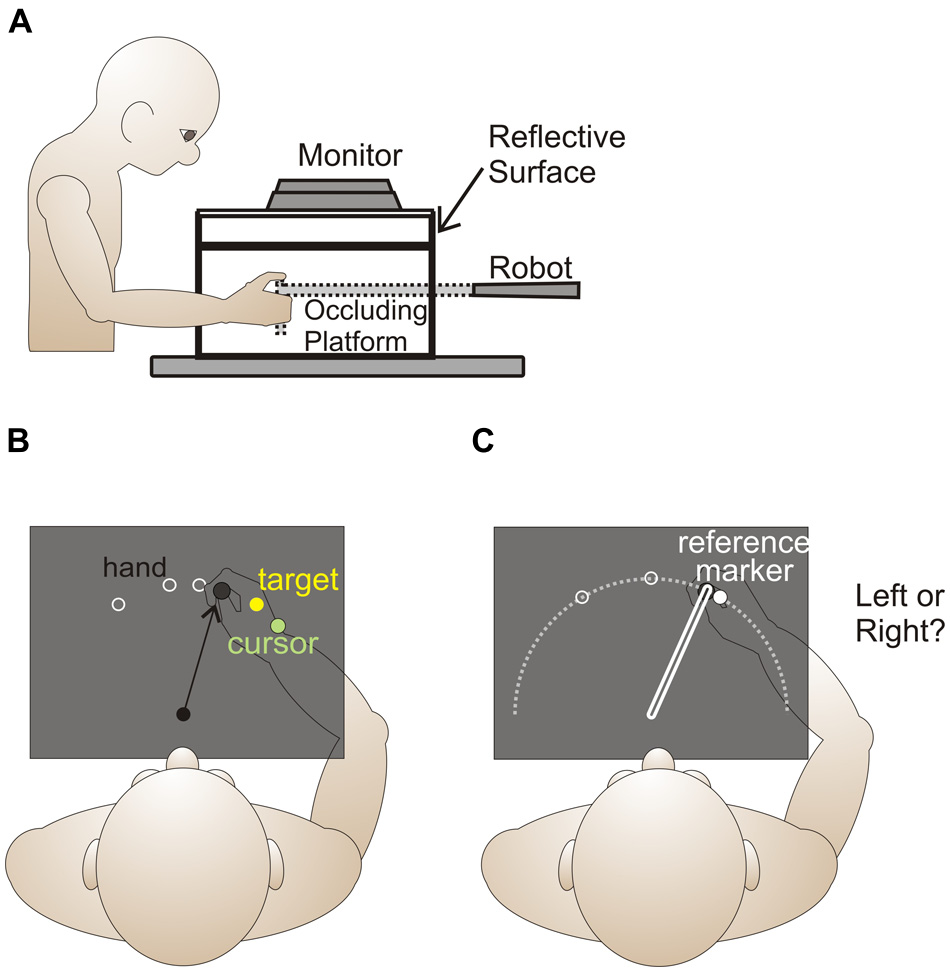
**Experimental setup and design.** Side **(A)** and top **(B,C)** view of experimental setup. **(B)** For both reaching training trials and no cursor reach trials, reach target locations, 1 cm in size, (locations indicated by the white rings) were 10 cm from the home position (shown as a black circle), and were 5° and 30° left and right of the body midline. In reach training trials, visual feedback of the unseen hand was provided by displaying a green cursor at the end of an initial reach in order that subjects could obtain the target (yellow circle). During the first rotated training block, the green cursor, representing the hand, was gradually rotated to 30° clockwise, and remained at this magnitude for the rest of the task and throughout the remaining two blocks. **(C)** Hand-proprioceptive estimate task. Trials started from a home position, which was illuminated by a 1 cm dot for 500 ms. After the home position disappeared, subjects pushed their hand out along a robot-guided constrained linear path (white rectangle on right) to a location on the white arc (not shown to subjects) relative to 1 of 3 possible reference markers (locations indicated by white circles) 10 cm from the home position. The reference markers, which appeared only after the hand had finished its outward movement, were 1 cm in diameter and located at 0° and 30° left and right of the body midline. Subjects were required to indicate if their hand was left or right of the reference marker.

**Figure 1A** provides a side view of the experimental set-up for the current and previous study. Subjects were seated in a height-adjustable chair in order that they could comfortably view and reach to all targets and reference markers presented on an opaque, reflective surface while grasping the vertical handle of a two-joint robot manipulandum (Interactive Motion Technologies) with their right hand. The position of the robot handle was recorded at a sampling rate of 50 Hz and had a spatial accuracy of 0.1 mm.

Installed 17 cm above the robot arm was a reflective surface onto which visual stimuli were projected from an LCD monitor (Samsung 510N, refresh rate 72 Hz). The reflective surface was positioned so that targets and reference markers projected onto the surface appeared to lie in the same horizontal plane as the unseen robot manipulandum. All natural light was blocked from the room, the room lights were dimmed, and subject’s view of their right hand and the manipulandum was occluded by the reflective surface and a black cloth that covered their right shoulder to the reflective surface.

### GENERAL PROCEDURE

**FIGURE 2:**
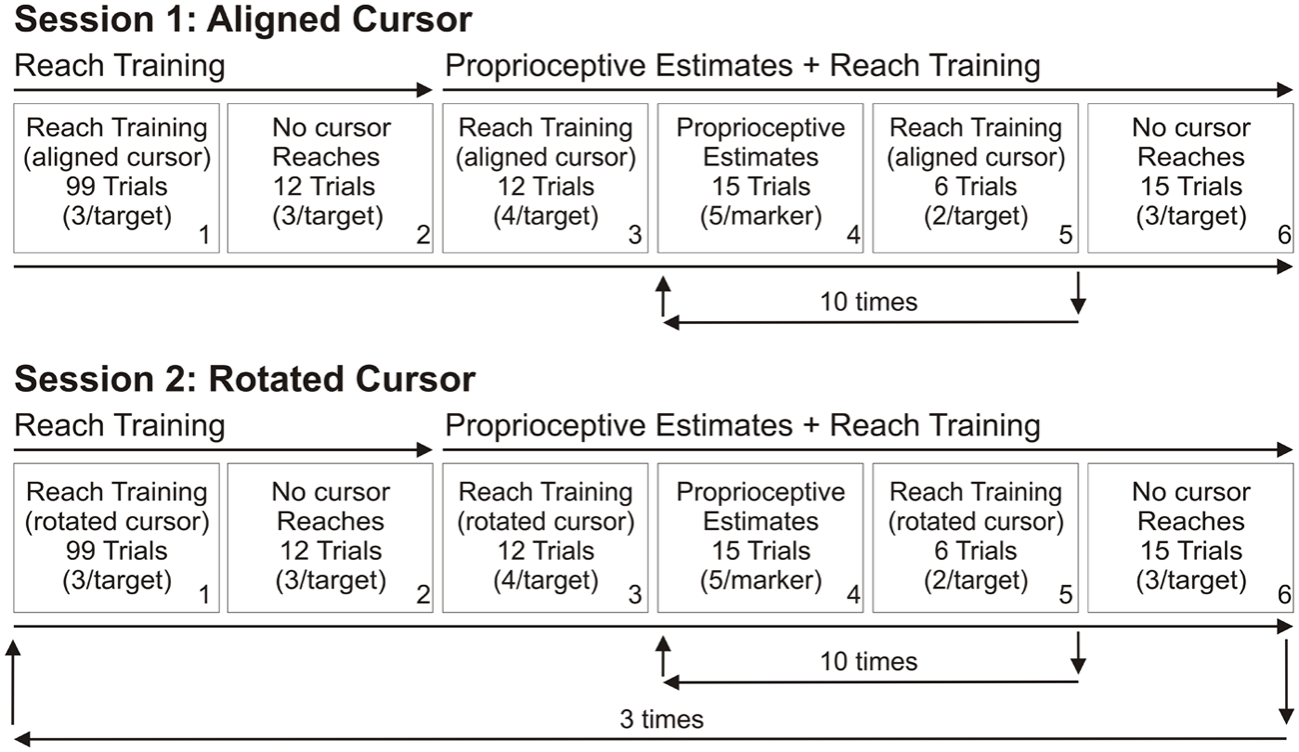
**Order of the tasks completed in the two testing sessions**. Each session was completed on separate days. Top row: Session 1. In the first testing session, subjects reached to targets with terminal hand-cursor feedback such that the cursor was aligned with the hand (Box 1). This reach training was followed by no cursor reach trials (Box 2). Afterwards, proprioceptive estimate trials were interleaved with further reach training trials. This sequence was repeated a total of 10 times (Boxes 4 and 5). The session ended with another set of no cursor reach trials (Box 6). Session 1 served as a baseline. Bottom row: Session 2. In the second testing session, the tasks (Boxes 1–6) were similar to those in Day 1, except the terminal cursor feedback was gradually rotated 30° CW from their actual hand position, reaching its full rotation of 30° by the 41st trial, and remaining at this rotation for the remainder of the trials (Box 1) and subsequent reach training trials (Boxes 3 and 5) and the additional training sets. These tasks (Boxes 1–6) constitute one block, and were repeated twice more for a total of three blocks.

To determine the effect of visual feedback quality on reach adaptation and changes in proprioceptive sense of hand position, we had subjects reach to targets with terminal visual feedback of their hand position, and compared their performance with subjects who had previously participated in a similar study in which continuous visual feedback of the hand was provided (Salomonczyk et al., 2011). For the terminal feedback group, during reach training trials, subjects were only shown the hand-cursor at the end of their ballistic reach movements, while subjects in the continuous feedback group were first shown the hand-cursor after the hand had traveled 4 cm from the home position toward the target (located 10 cm from the home position), up until the cursor acquired the visible target. Following the reach training tasks, both groups then reached to the same targets without any hand-cursor feedback and performed a proprioceptive estimation task. Both groups performed two different testing sessions on two separate days (**Figure 2**). For session one, reach training trials involved a cursor that was aligned with the unseen reaching hand to provide baseline measures of performance (**Figure 2**, top row). For session two, the cursor was rotated during reach training trials, and the reach training, no cursor reaches and proprioceptive estimate tasks were repeated three times in succession (**Figure 2**, bottom row).

#### Task 1: Reach training

In the reach training task (**Figures 1B** and **Figure 2**, Boxes 1, 3, and 5), subjects reached to a visual target (yellow circle, 1 cm in diameter) from the home position using the robot manipulandum. Four reach targets were radially located 10 cm from the home position: 30° counterclockwise (CCW), 30° clockwise (CW), 5° CCW, and 5° CW of the body’s midline (**Figure 1B**). Visual feedback was provided in the form of a hand-cursor (green circle, 1 cm in diameter) that indicated the reach end position (terminal feedback). The cursor was aligned with the actual hand position in the first testing session (**Figure 2**, top row) and gradually rotated to 30° CW relative to hand position during the first block of the second testing session (bottom row). Subjects began their reaches from a home position that was approximately 40 cm in front of them and aligned with their body midline. The home position was not illuminated during reach training trials. At the end of each reach trial, visual feedback was eliminated, and subjects returned their hand to the home position along a robot-established linear route (similar to Salomonczyk et al., 2011). If subjects attempted to move outside this linear route or grooved wall, a resistance force was generated [proportional to the depth of penetration with a stiffness of 2 N/mm and a visual damping of 5 N/(mm/s)] perpendicular to the grooved wall (also in (Henriques and Soechting, 2003; Cressman and Henriques, 2009, 2010; Cressman et al., 2010; Jones et al., 2010). Trial order was pseudo-randomized such that subjects reached to each of the two peripheral targets and one of two of the peri-central targets prior to any target repeating. Subjects completed one set of 99 reach trials with the aligned-cursor in the first testing session (**Figure 2**, Box 1, top row) and three sets of 99 reach trials with the rotated-cursor in the second training session (Box 1, bottom row). In the first set of the rotated reach training trials, the cursor rotation was gradually introduced by rotating the cursor 0.75° CW relative to the hand each trial, until the maximum rotation of 30° CW was achieved on the 41st trial. This 30° CW rotation was maintained for all subsequent reach training.

During reach training trials with terminal feedback, the hand-cursor was not illuminated until the initial reach movement was complete, i.e., when the velocity of the hand was less than or equal to 3 mm/s for 0.5 s. At this point, the hand-cursor appeared in order to provide subjects with a visual representation of their hand location relative to the target at the end of their initial ballistic motion. After the hand-cursor appeared, subjects were told to move the illuminated hand-cursor to the visible target, and the trial ended when the hand-cursor’s center and the target’s center were within 0.5 cm of each other. We do not expect that the post-reach motion to target had a significant impact on no cursor reaches; Tseng et al. (2007) compared continuous feedback reach adaptation and aftereffects between subjects who were either permitted to make online corrective movements or not, and no differences were found between groups. On average, subjects moved approximately 2.4 cm while seeing the hand-cursor across all reach training trials. In the infrequent case when subjects managed to obtain the target in the first ballistic motion, the trial ended immediately. At the end of the trial, no visual feedback was provided from the hand-cursor, the target disappeared, and subjects returned their hand to the position along a robot-generated, linear route. In contrast, for subjects training with continuous feedback, the hand-cursor was first displayed once the hand had moved 4 cm from the home position. The hand-cursor then remained visible until subjects acquired the target (Salomonczyk et al., 2011). Thus, subjects who experienced continuous visual feedback experienced real-time feedback about their unseen hand’s position in the workspace during their first ballistic motion.

Prior to the reach training task in the first testing session, subjects in the terminal feedback group were given a practice session of 20 reach training trials with the aligned hand-cursor visible during the entire reach so that subjects could become accustomed to the apparatus and reach task prior to introducing terminal visual feedback. In the continuous feedback condition, there were no preceding practice trials.

#### Task 2: No cursor reaching

In the no cursor reaching task (**Figure 2**, Boxes 2 and 6), subjects reached to the same visible targets but without visual feedback of the hand-cursor. After subjects held their end position for 0.5 s, the target disappeared, and subjects’ hands were again guided back to the home position by a linear grooved path. We calculated reach aftereffects, by subtracting reach endpoints made without a cursor after aligned-cursor training (top row) from those produced after rotated-cursor training (bottom row). Subjects reached to four visual targets three times (Box 2), and to the same four targets plus one additional target at 0° (i.e., body midline or center) following proprioceptive estimate trials with interleaved reach training (Box 6). This second set of no cursor trials was to assess whether the aftereffects, and thus, reach adaptation decreased or decayed during the proprioceptive estimate test described below.

#### Task 3: Proprioceptive estimates

**FIGURE 3:**
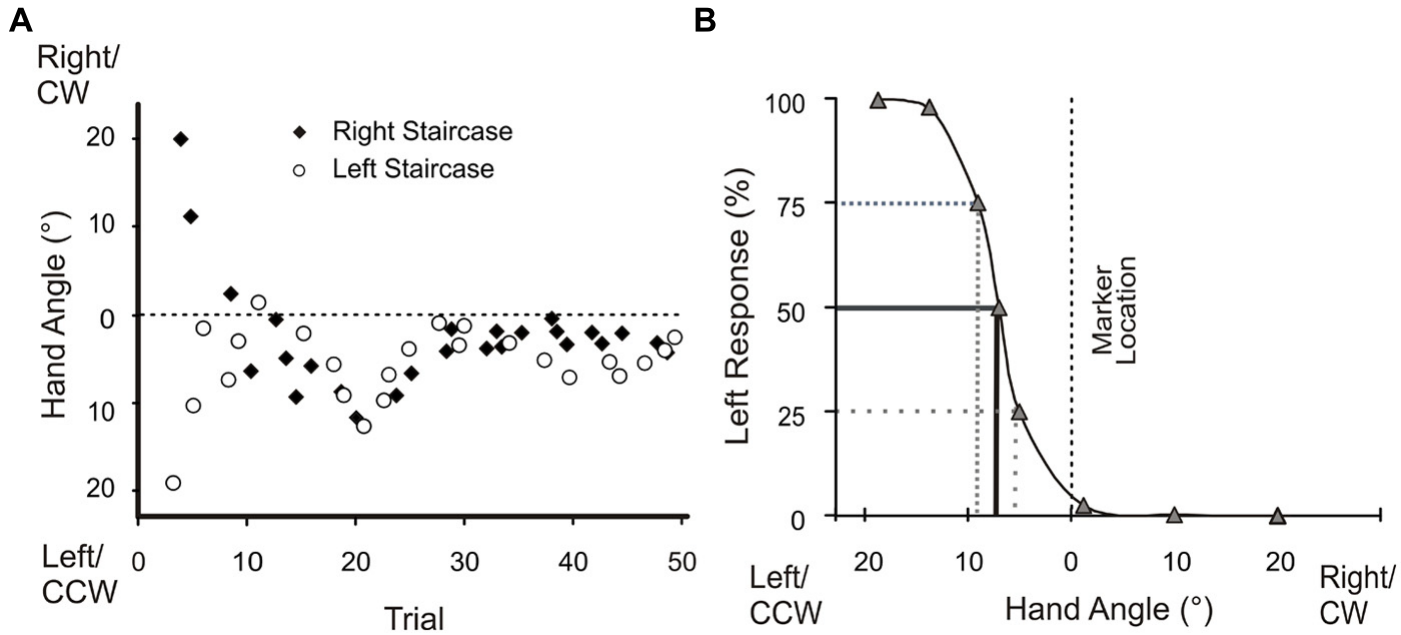
**Angular hand position during proprioceptive estimate trials and percentage of left responses for the 0° visual reference marker for a single subject**. **(A)** The left and right staircases began with a subject’s hand placed 20° from either side of the reference marker (dotted line). These adaptive staircases progressively converged over successive trials. **(B)** A logistic function was fitted to a representative subject’s data to define bias; where bias is the probability of responding left 50% of the time.

Proprioceptive estimate trials (**Figure 2**, Box 4) began with subjects holding their hand at the home position. The home position, indicated by a green, 1 cm diameter circle, was illuminated for 0.5 s. After the home position disappeared, subjects were instructed to push their hand outward along a robot-constrained, 10 cm long, linear path (**Figure 1C**, elongated rectangle). When a subject’s hand arrived at the end of the path, a reference marker (yellow, 1 cm-diameter circle) appeared. Subjects were instructed to make a two-alternative forced-choice decision regarding whether they felt that their unseen hand was to the left or right of this reference marker. Following their response, subjects returned their hand to the start position using the same robot-generated, linear path and began the next trial. The reference markers were located 30° CCW, 30° CW or 0° relative to the body midline (**Figure 1C**, white and open circles). Subjects’ hand position relative to each reference marker was adjusted over the course of 50 trials using an adaptive staircase algorithm (Kestin, 1958; Treutwein, 1995), as previously described in our other studies (Cressman and Henriques, 2009, 2010; Jones et al., 2010; Salomonczyk et al., 2011). As in Salomonczyk et al. (2011), there were two staircases per reference marker, each starting at 20° either left (CCW) or right (CW) of the reference marker (**Figure 3A**). As outlined by Cressman and Henriques (2009), the two staircases were adjusted individually and randomly interleaved.

Proprioceptive estimate trials were interleaved with reach training trials (**Figure 2**, Boxes 4 and 5). Fifteen proprioceptive estimate trials and six reach training trials (with either an aligned or rotated cursor) immediately followed these initial reach training trials (**Figure 2**, Boxes 4 and 5). A set of 15 proprioceptive estimate trials and 6 reach training trials was completed 10 times, and then subjects performed 15 no cursor reaching trials. Thus, there were a total of 150 proprioceptive estimate trials per block.

### TESTING SESSIONS: ALIGNED AND MISALIGNED BLOCKS

The three aforementioned tasks were arranged in blocks within testing sessions that were completed on two days, between 24 h and 30 days apart. Each block consisted of 99 trials of reach training (**Figure 2**, Box 1), no cursor reaches (Box 2), proprioceptive estimate trials intermixed with further reach training trials (Boxes 3–5), and ended with a second set of no cursor (aftereffect) reaches (Box 6). Only one block was completed in the first testing session, where the cursor was aligned with the hand in reach training trials, and the no cursor reach errors and proprioceptive estimates served as a baseline for future rotated cursor blocks. The second day of testing consisted of three blocks which were performed in succession, as it was unknown whether reach adaptation or shifts in felt hand position following training with terminal feedback would be evident after only one block, (as was the case for continuous feedback) or would require a second or third block of training. Moreover, it was unclear if these changes would increase in size with each set of reach training. The testing sessions were identical to those in the continuous visual feedback study (Salomonczyk et al., 2011).

### DATA ANALYSIS

#### Reaches: motor adaptation

Our main analysis was to determine if open-loop reach errors (i.e., aftereffects) following rotated-cursor training differed from those following aligned-cursor training and if aftereffects following each set of 99 trials with the rotated cursor differed from one another. We also compared these differences or aftereffects across the two sets of no cursor reaches within each block (epoch 1 and epoch 2) to determine if the aftereffects decayed following proprioceptive estimates interleaved with reach training. To examine reach errors, we analyzed the endpoint angle errors and the angle of the hand at peak velocity (PV) in the no cursor reach trials. Endpoint errors were defined as the angular difference between a movement vector (the linear path from the home position to movement endpoint) and the reference vector (the linear path joining the home position to the target). PV angle was defined as the difference in angle between a movement vector, which joined the home position to the point at which the hand reached PV, and the reference vector. For both endpoint errors and angle at PV, we conducted a 4 block (aligned 1 vs. rotated 1 vs. rotated 2 vs. rotated 3) by 2 epoch (post-reach training vs. post-proprioceptive estimates with interleaved reaching) by 4 target location (30° left vs. 30° right vs. 5° left vs. 5° right) RM-ANOVA for the terminal feedback group. In order to determine if additional training with rotated terminal feedback yielded any increase in aftereffects over successive blocks, we calculated reach aftereffects by subtracting the no cursor reaches for the aligned block from each of those in the three rotated blocks, and then ran another three-way ANOVA but this time with only three blocks (rotated 1–3). Likewise, we used reach aftereffects to compare these changes in movements for the terminal feedback and continuous feedback group, using a mixed ANOVA with visual feedback type (terminal versus continuous) as a between-subjects factor and block (rotated 1 vs. rotated 2 vs. rotated 3) and epoch (post-reach training vs. post-proprioceptive estimates with interleaved reaching) as within subjects factors.

#### Proprioceptive estimates of hand position

We examined the influence of training with terminal hand-cursor visual feedback on proprioceptive estimates of hand position. For each subject, we fit a logistic function to his or her responses for each reference marker (**Figure 3B**). From the logistic function we determined the subject’s bias, which is an estimate of the subject’s accuracy of their sense of felt hand position (Cressman and Henriques, 2009, 2010). Bias was represented by the point at which subjects responded “left” (and “right”) 50% of the time (Cressman and Henriques, 2009, 2010; Jones et al., 2010; Salomonczyk et al., 2011). We compared these estimates of felt hand position relative to reference markers after aligned-cursor training (baseline) with those after misaligned-cursor training.

Bias was analyzed in a 4 block (aligned 1 vs. rotated block 1 vs. rotated block 2 vs. rotated block 3) by 3 reference marker location (30° CCW, 0°, 30° CW) RM-ANOVA. This was followed by another ANOVA where we compared the changes in sense of felt hand position across additional rotated-training blocks by subtracting biases from the aligned session from those biases measured following each rotated set, so that the number of training blocks was reduced to three. These changes were then compared to changes in sense of felt hand position following reach training with continuous visual feedback of the hand in a 2 by 3 mixed ANOVA with visual feedback type (terminal and continuous) as a between-subjects factor and block as a within subjects factor.

For all ANOVAs, differences with a probability of less than 0.05 were considered significant and pairwise comparisons were Bonferroni corrected. We report Greenhouse–Geisser corrected *p*-values when required.

## RESULTS

### MOTOR ADAPTATION

**FIGURE 4:**
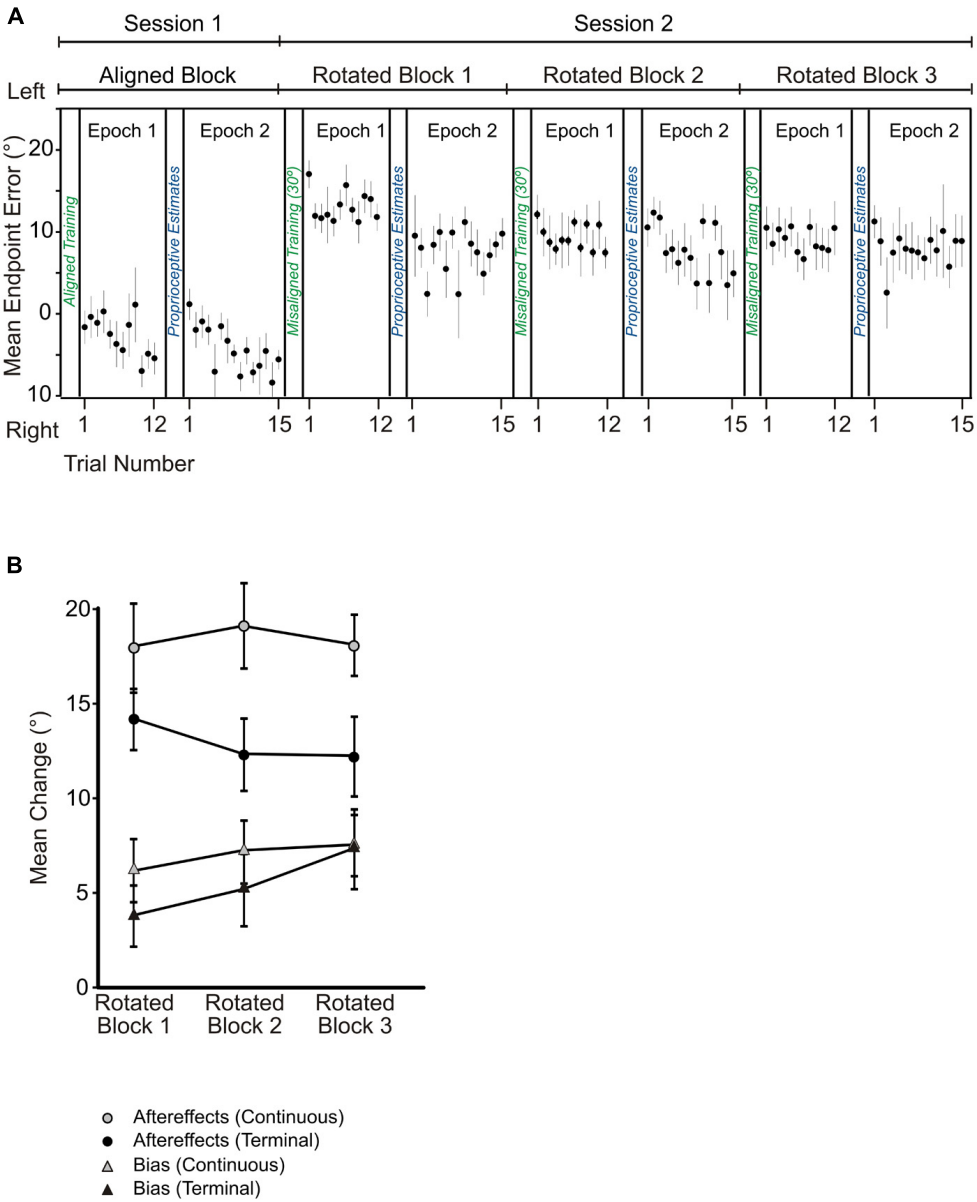
**(A)** Angular reach endpoint errors plotted across no cursor trials in the aligned and rotated blocks following terminal feedback training. Black circles are averaged across subjects for these no cursor reaches, and error bars represent standard error of the mean. **(B)** Angular changes in no cursor reaches (i.e., reach aftereffects, indicated by circles) and proprioceptive biases (triangles) across the three blocks of rotated reach training relative to performance in the first testing session with the aligned hand-cursor. Dark symbols indicate mean performance from the terminal feedback experiment while gray symbols represent those from the continuous feedback experiment (Salomonczyk et al., 2011). Mean changes in degrees were averaged across subjects and across target/reference marker locations. Error bars represent standard error of the mean.

Subjects reached to targets with an average movement time of 1.18 ± 0.34 s (SD) and an average PV of 15.85 ± 9.52 cm/s (SD) in the no cursor reaches. In Salomonczyk et al. (2011), the average movement time was 1.78 ± 0.8 s (SD) and the average PV was 16.4 ± 5.9 cm/s (SD). Mean reach endpoint errors for trials performed after aligned-cursor training were 3.73° to the right of the target, as illustrated by the first two sets of reach endpoints plotted in **Figure 4A** (labeled session 1). These open-loop reaching errors (prior to adaptation) indicate that subjects were moderately accurate with their reaches to targets even when they lacked visual feedback pertaining to their hand position. These reach errors were a bit more shifted than those observed in the continuous feedback study: in our previous study, these errors were 0.75° to the right of the target (Salomonczyk et al., 2011).

We compared these open-loop reaches following training with an aligned cursor with those following rotated-cursor training, as illustrated in **Figure 4A**, which plots these reaches across trials for the aligned block and the three rotated blocks for the terminal feedback group. We found a substantial shift in the direction that subjects reached after training with both terminal and continuous feedback, the extent of which is shown by the black and gray circles in **Figure 4B**. For terminal feedback training, the no cursor reaches deviated significantly leftwards compared to the reaches following the aligned-cursor training block, *F*(3,30) = 36.97, *p* < 0.001, and this was true following all three blocks of rotated-cursor training: aligned cursor block-rotated cursor block: rotated block 1 = 14.1° (*p* < 0.001); rotated block 2 = 12.06° (*p* < 0.001); rotated block 3 = 11.84° (*p* = 0.001). The no cursor reaches relative to baseline (i.e., reach aftereffects) for the terminal feedback group (**Figure 4B**, black circles) were slightly smaller, by roughly 5.8° across rotated blocks than those found for the continuous feedback group (gray circles), *F*(1,19) = 4.5, *p* = 0.047. As reported in Salomonczyk et al. (2011), the no cursor reaches were also significantly different between the aligned block and the three rotated blocks when subjects used continuous feedback. We also found that further rotated training with terminal feedback (the additional two blocks) did not lead to substantially larger aftereffects, *F*(2,20) = 2.21,*p* = 0.136. The same was true for subjects receiving continuous feedback (Salomonczyk et al., 2011; **Figure 4B**, gray squares).

When we compared the terminal feedback aftereffects (i.e., change in no cursor reaches relative to baseline performance) made soon after reach training (epoch 1) with the aftereffects completed after proprioceptive estimates (epoch 2), we found no significant difference across the three blocks, *F*(1,10) = 1.67, *p* = 0.22. Likewise, no changes in epoch were found for the continuous feedback group (Salomonczyk et al., 2011). Thus, subjects reached with similar errors before and after completing the proprioceptive estimate trials.

We found a similar pattern of results for changes in the angular reach deviation at PV, as we did for the angular endpoint errors described above for the terminal feedback group. Directional errors at PV were significantly more leftward following all rotated reach training blocks compared to the aligned training block, [*F*(1.461,14.609) = 19.16, *p* < 0.001], in that all comparisons of these no cursor reaches between the aligned training block and each of the three rotated blocks were significantly different (*p* < 0.01). When comparing reach aftereffects, for the most part, the angular deviations at PV closely resembled those of the endpoints (within 2°) for the terminal feedback group. This was different than the continuous feedback group, where the angle at PV deviated from the endpoint error by 5°, suggesting that these open–loop reaches were much straighter in the terminal feedback group than in the continuous feedback group. Overall, there was no change over rotated training blocks, thus additional rotated training had no significant impact on PV angle.

### BIAS

**FIGURE 5:**
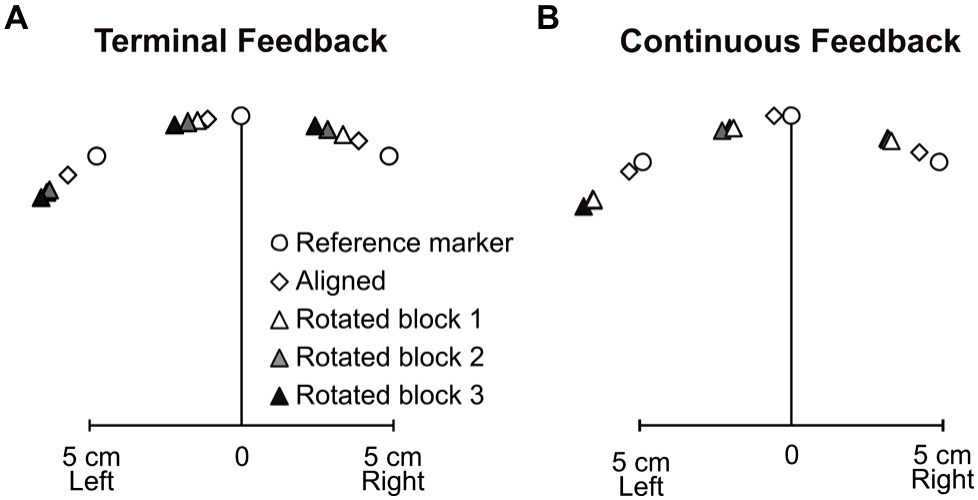
**Mean 2-D proprioceptive biases for the **(A)** terminal feedback experiment and **(B)** continuous feedback experiment (adapted from Salomonczyk et al., 2011).** Subjects estimated their hand position relative to reference markers (open circles) following aligned hand-cursor feedback training (open diamonds) and rotated hand-cursor training (first rotated block: white triangles; second rotated block: gray triangles; third rotated block: dark triangles).

Next, we wanted to determine if adapting to a rotated cursor with terminal feedback also led to similar changes in felt hand position, i.e., proprioceptive recalibration, as has been seen after training with continuous visual feedback of the hand-cursor. **Figure 5A** displays the three reference marker locations (circles), average biases following aligned-cursor training (diamonds) and rotated-cursor training (triangles) when terminal feedback was provided. Each successively darker triangle represents subject’s estimates of felt hand position relative to the reference marker for rotated blocks 1, 2, and 3. **Figure 5B** uses the same schematic to illustrate the results under continuous feedback conditions (Salomonczyk et al., 2011). In the terminal feedback condition, for the aligned block, felt hand positions were slightly left of the reference markers, specifically 7.27° left of the reference marker. This leftward bias has been previously observed in our lab and is due to a hand bias (Jones et al., 2010); this hand bias was also observed in the continuous feedback condition (Salomonczyk et al., 2011), where the average bias across subjects and reference markers for the aligned block was 5.1° leftward.

For terminal feedback, we see that each rotated block yielded estimates of felt hand positions that were successively further left of the reference markers and the estimates after training with an aligned hand-cursor, consistent with the direction of the visuomotor distortion (**Figure 4B**), black triangles). There was a main effect of training block among the aligned and three rotated training blocks, *F*(3,30) = 8.62, *p* < 0.001. Thus, we next assessed whether biases after each rotated set were significantly shifted relative to those following the aligned-cursor training. We found that although biases were not significantly shifted for the first rotated block, they were for the second and third rotated blocks relative to the aligned block: rotated block 1 – aligned, 3.39° (*p* = 0.404); rotated block 2 – aligned, 5.12° (*p* = 0.035); rotated block 3 – aligned, 7.41° (*p* = 0.008). Additionally, the change was much larger, by 4.01°, in the last rotated block compared to the first rotated block (*p* = 0.029), suggesting that more practice with terminal feedback led to greater proprioceptive recalibration (illustrated by the increasing height of the black triangles across blocks in **Figure 4B**). This was not the case for the continuous feedback group (Salomonczyk et al., 2011), where the significant change in bias saturated after the first set of rotated training (**Figure 4B**, gray triangles).

Interestingly, we found that the overall size of the change in felt hand position was similar across the terminal and continuous feedback groups, in that there was no significant difference in changes in bias for the terminal feedback and continuous feedback groups, *F*(1,19) = 0.56, *p* = 0.46. Although Salomonczyk et al. (2011) did not find a significant difference across the three blocks of rotated training, when we looked at the change in bias across the three rotated blocks for the terminal feedback group, we found that they did significantly differ as explained above. Thus, both feedback groups reached a similar level of change in felt hand position by the end of the three training blocks.

### MOTOR ADAPTATION AND PROPRIOCEPTIVE RECALIBRATION

To better compare changes in reaches (aftereffects) to changes in felt hand position, we ran a linear regression to see whether changes in felt hand position depended on changes in reach aftereffects. As consistent with our previous studies (Cressman and Henriques, 2009; Salomonczyk et al., 2011, 2012, 2013), we found no significant relationship between the changes (*p* = 0.17, *R*^2^ = 0.06), although as usual the change in felt hand position was much smaller than the reaching aftereffects for the two feedback groups (**Figure 4B**). More importantly, we found that despite significantly smaller reach aftereffects following terminal feedback training, compared to continuous feedback training, the overall change in felt hand position was similar between the two feedback groups, at least by the third block. Again, this suggests that the sensory changes are not directly related to motor changes.

## DISCUSSION

The main goal of the present study was to examine whether terminal feedback experienced during reach training affects our subsequent estimates of felt hand position. Subjects reached to three targets for a total of 99 trials with visual feedback of their hand rotated 30° CW relative to hand movement, in three reach training blocks. Visual feedback was only provided at the end of the primary movement. After each training set of 99 trials, subjects reached to the same targets without a cursor, and then estimated the position of their trained, unseen hand relative to reference markers at similar locations. On average, we found that subjects who experienced terminal visual feedback both adapted their reaches and recalibrated their felt hand position. Mean reach aftereffects approached 13° after the first rotated block, and were maintained at that level even after two additional training blocks. Sense of felt hand position was also recalibrated by 3.4° after the first training block; however, changes in felt hand position increased further and significantly to 7.41° by the third reach training block. Compared to another group of subjects who experienced continuous feedback (Salomonczyk et al., 2011), subjects experiencing terminal feedback appeared to adapt their reaches less (by about 33%) over the three training blocks, but their sense of felt hand position, although initially shifted less than subjects in the continuous feedback group, reached a comparable level by the third training block. Thus, we found that terminal feedback was sufficient to drive reach adaptation, and despite subjects seeing the visual representation of their hand only for a limited time at the end of the movement, they successfully recalibrated their felt hand position to a level comparable to subjects with continuous feedback training after additional training.

### ROLE OF VISUAL FEEDBACK QUALITY IN REACH ADAPTATION

In the current study, we provided three sets of reach training trials in order to determine how long it took for reach adaptation to saturate when terminal feedback was provided (by the end of the third training set, subjects had reached to each of the targets 99 times). Surprisingly, our extra training trials did not lead to increased aftereffects over successive training blocks, such that aftereffects following the first 99 training trials were not significantly different from those found after all 297 trials (reach adaptation equal to ∼13°). This early saturation of reach adaptation is similar to our previous results in which subjects completed the same three training blocks to the same targets with a continuously visible rotated cursor (reach adaptation equal to ∼18.44°; Salomonczyk et al., 2011). Moreover, we have seen reach adaptation saturate quickly in an earlier study of ours (Wong and Henriques, 2009), where we had subjects reach with a rotated cursor to similar targets for at least 200 trials each day for five consecutive days. Thus, we have shown that increased training neither helped nor decreased the discrepancy in the extent of motor adaptation between terminal and continuous feedback conditions. Also, terminal feedback resulted in smaller reach aftereffects, compared to continuous feedback. These smaller aftereffects were not due to decay over the open-loop reach trials, since no cursor reaches were constant within a block.

The reach aftereffects we found following terminal feedback training were about 66% of the size of those found following continuous feedback in our earlier study (Salomonczyk et al., 2011), and reflect significant reach adaptation. These results differ from those of Hinder et al. (2008) and Shabbott and Sainburg (2010) who found no significant reach aftereffects. Their aftereffects were based on reaches produced when the aligned cursor was reintroduced (rather than removed, like in this study), and training feedback involved not just cursor endpoint alone, but the entire hand path display (what they called knowledge of results – KR). However, our results are consistent with the majority of studies that used endpoint feedback during training and measured aftereffects based on no cursor reaches (which would be associated with smaller washout). For instance, van der Kooij et al. (2013) and Taylor et al. (2014) both showed significant, yet smaller, reach aftereffects following terminal feedback training compared to continuous training. For example, van der Kooij et al. (2013) found significant changes in open-loop reaches following training with terminal feedback, or what they called realignment of the unseen hand, and these changes were about one third smaller than those produced by subjects who trained with continuous feedback. Again, the distortion they used, although abruptly introduced, was rather small (5° deviation relative to the cyclopean eye). In a recent paper by Taylor et al. (2014), following terminal feedback training with an abrupt, 45° cursor rotation, reach aftereffects were roughly 66% the size of those produced following training with abrupt continuous feedback. During reach training, some subjects verbally reported which target they were going to aim for prior to each reach – the instruction groups. The reach aftereffects for these subjects in the instruction group did not significantly differ from those produced by subjects who reached without making a verbal report, following either continuous or terminal feedback training. In addition, the relative magnitude of these reach aftereffects in their study (terminal vs. continuous) is similar to that found by van der Kooij et al. (2013) and the current study. And while Taylor et al. (2014) suggest that differences in reported aiming direction during training for the instruction groups indicates that terminal feedback resulted in greater explicit learning compared to continuous feedback, our results neither support nor refute this interpretation since our distortion was gradually introduced, and thus less likely to engage explicit learning processes. Interestingly, Bernier et al. (2005) showed that following training with continuous feedback, aftereffects washed out quickly while those following training with KR were initially large and did not washout. Like us, Bernier et al. (2005) also gradually introduced a rather small visual perturbation and had subjects reach 80 times to each of three nearby targets. Likewise, in Izawa et al. (2012), a gradually introduced and small, 8° cursor rotation led to near equivalent aftereffects in the direction of the trained target (although generalization to novel but proximal targets was about 50% smaller). Thus, taken together, these studies suggest that significant reach aftereffects arise after training with terminal feedback, when assessed by open-loop reaches.

Previous results of ours suggest that when the cursor feedback is continuous during training, there is no difference in aftereffects regardless of whether the 30° cursor rotation was introduced gradually or abruptly (Salomonczyk et al., 2012). Klassen et al. (2005) also found no difference between abrupt and gradual rotated training (for a 30° rotation) when they measured retention of adaptation a day later. However, reach aftereffects have been found to be smaller following abrupt cursor rotation compared to a gradual one when the perturbation is particularly large [e.g., 90°; Kagerer et al., 1997; Buch et al., 2003; *N.B.* Buch et al. (2003) only found this for their older subject group]. Thus, it is possible that for more challenging perturbations, including perhaps ones involving terminal feedback, the manner in which the distortion is introduced may influence reach aftereffects. In contrast, given that studies using an abrupt perturbation (van der Kooij et al., 2013; Taylor et al., 2014) and those using a gradually introduced perturbation (Izawa and Shadmehr, 2011; and the current study) found that changes in open-loop reaches after training with terminal feedback were at least two-thirds the size of those produced following training with continuous feedback, the manner in which the distortion is introduced may make little difference when the distortion is small (e.g., less than 45°).

### THE EFFECT OF TRAINING WITH TERMINAL FEEDBACK ON HAND PROPRIOCEPTION

In our study, we derived subject’s sense of felt hand position with a task that does not require goal-directed reaches, by asking subjects to report the location of their (robot-guided) felt hand position relative to a reference marker (Cressman and Henriques, 2010, 2011; Cressman et al., 2010; Salomonczyk et al., 2011, 2012; Clayton et al., 2013; Salomonczyk et al., 2013; Mostafa et al., 2014). We found that subjects recalibrated their felt hand position following rotated hand-cursor training, even after training with only terminally altered feedback of their hand. However, this proprioceptive shift only achieved significance after the second block of reach training, and continued to increase in size during the third and final block. By this final block of rotated terminal feedback training, subject’s shift in felt hand position was comparable to shifts in felt hand position experienced by subjects in the continuous feedback condition. With continuous feedback, Salomonczyk et al. (2011) found that additional training, beyond the first block of 99 trials, did not lead to further recalibration following a 30° rotation; however, gradually increasing the cursor rotation (up to 70°) did lead to larger changes in felt hand position (as well as reach aftereffects). This change in felt hand position following rotated continuous feedback training was similar whether the cursor was gradually or abruptly introduced (Salomonczyk et al., 2012). It is unknown whether introducing the terminally misaligned cursor abruptly would have a similar effect on proprioceptive recalibration.

In addition to changes in felt hand position, it has recently been shown that visuomotor adaptation leads to changes in estimating the sensory consequences of self-guided hand movements (Synofzik et al., 2008; Izawa and Shadmehr, 2011; Izawa et al., 2012). That is, people mislocalize the direction by which they move their unseen hand across a landmark following visuomotor adaptation to a rotated cursor. To look at this, Izawa and Shadmehr (2011) measured both reach aftereffects and hand localization errors under different feedback conditions, including training with continuous and terminal misaligned feedback of the hand that was gradually introduced. They found that reach aftereffects were equivalent, at least in the direction of training (generalization to novel directions was smaller for terminal feedback training than for continuous), and the errors in predicting the consequences of these movements (the hand localization errors) were about 30% smaller following training with terminal feedback compared to continuous feedback. Together, these studies show that changes in felt hand position and sensory prediction errors follow different patterns depending on whether there was continuous or terminal feedback.

### INDEPENDENCE OF REACH ADAPTATION AND PROPRIOCEPTIVE RECALIBRATION

Our results, along with those from prior studies from our lab and others, suggest that changes in reaches and changes in felt hand position following training with altered visual feedback of the hand are independent of each other. First, the point in training by which maximum changes were achieved was different for the two feedback conditions, such that 99 training trials were needed for motor adaptation to saturate, and 297 training trials were needed for changes in bias to reach maximum levels achieved in an earlier study. Similar to the results for continuous rotated feedback (Salomonczyk et al., 2011), we also found no significant correlation between the changes in reaches and hand proprioception. Results from related studies in our lab have also shown this lack of correlation, or different rates of change between motor adaptation and sensory consequences. Finally, and more convincingly, we have shown different patterns of generalization for reach aftereffects and changes in hand proprioception (Mostafa et al., 2014).

Along with results from our lab, results from studies testing patients with cerebellar damage or using a force-field perturbation suggest this independence of motor and sensory changes following training with a rotated cursor. For example, in Izawa et al. (2012), while cerebellar patients adapted their reaches to a perturbation that was gradually introduced to the same extent as controls (similar reach aftereffects), patients showed smaller changes in what the authors called the predictive consequences of unseen hand movements; these were measured by having subjects reach with their unadapted hand, to the location at which they perceived their unseen adapted hand had previously moved. Moreover, Synofzik et al. (2008) found that while cerebellar patients did not learn to adapt their reaches to a cursor rotation that increased by 6° per trial (i.e., somewhat abruptly) as well as controls, they did recalibrate their estimates of their arm movements. However, similar to Izawa et al. (2012), this recalibration level seen in the patients was less than in the controls. In a force-field perturbation paradigm, Mattar et al. (2013) recently showed different patterns in the rate of adaptation and the rate of change in what they called the perceptual boundary of the adapted hand. In brief, the pattern of changes in motor adaptation and proprioceptive recalibration following training with terminal feedback in the current study add to the argument for motor adaptation and sensory recalibration’s independence.

## CONCLUSION

Following visuomotor adaptation using terminal visual feedback, subjects adapted their reaches and recalibrated their sense of felt hand position, but these changes were smaller than those for subjects who received continuous visual feedback. Based on the present results, we suggest that terminal feedback provides sufficient information for motor learning, even after only 99 trials (33 trials per target). But, while motor adaptation remained relatively stable after the first rotated training block, additional training was necessary for attaining maximal changes in felt hand position. This difference in rate of motor adaptation vs. proprioceptive recalibration provides further support for the proposal that motor adaptation and sensory recalibration are two processes that change concurrently, yet independently. At present, the current results suggest that the amount of visual feedback available influences the time required for proprioceptive recalibration.

## Conflict of Interest Statement

The authors declare that the research was conducted in the absence of any commercial or financial relationships that could be construed as a potential conflict of interest.
